# A sensitive label–free amperometric immunosensor for alpha-fetoprotein based on gold nanorods with different aspect ratio

**DOI:** 10.1038/srep09939

**Published:** 2015-04-24

**Authors:** Chunyang Zhou, Dali Liu, Lin Xu, Qingling Li, Jian Song, Sai Xu, Ruiqing Xing, Hongwei Song

**Affiliations:** 1 State Key Laboratory on Integrated Optoelectronics, College of Electronic Science and Engineering, Jilin University, 2699 Qianjin Street, Changchun, 130012, P. R. China; 2 The State Key Laboratory of Bioelectronics, Southeast University, 210096 P. R. China

## Abstract

A simple and accurate label–free amperometric immunosensor for α–fetoprotein (AFP) detection is developed based on gold nanorods (GNRs) with different aspect ratio and compared with gold particles (GNPs). The positively charged GNRs and GNPs due to the surface immobilized cetyltrimethyl ammonium bromide (CTAB) can adsorb the negatively charged AFP antibody (Ab) directly. The presence of the GNRs not only enhanced the immobilized amount of biomolecules, but also improved the electrochemical properties of the immunosensor. With the aid of GNRs, the electrochemical signal was greatly enhanced in comparison with GNPs. Under optimal conditions, the proposed immunosensor could detect AFP in a linear range from 0.1 to 200 ng/mL with a detection limit of 0.04 ng/mL (signal–to–noise ratio = 3), and it also possessed good reproducibility and storage stability. Moreover, the detection of AFP in five human serum samples also showed satisfactory accuracy. The proposed methodology was potentially attractive for clinical immunoassay.

Cancer biomarker is an important risk indicator to establish the presence of a disease or to monitor the efficacy of a drug therapy. Generally, it refers to a certain protein concentration measured in blood or tissue in clinical diagnosis. As a significant biomarker, AFP is an acid glycoprotein with the molecular weight of 69000^1^,^2^,^3^,^4^, which exists in fetal development of early liver and yolk sac, and it gradually disappears so soon after the baby is birth. In the normal sense, the level of AFP concentration in healthy adult is less than 25 ng/mL^2^,^5^,^6^,^7^, whereas the value may exceed 400 ng/mL in case of pathological changes of liver, such as liver cancer^8^.

Accordingly, numerous immunological methods for determining the concentration of AFP have been described^1^,^6^,^9^,^10^. Now, the classic enzyme–linked immunosorbent assay (ELISA) is one of the most widely used methods for determination AFP in biological fluids based on spectrophotometric reading, however, it suffers the drawbacks of time–consuming, the short shelf life of the I–label antibody, and the narrow dynamic range^2^,^9^,^11^. As a promising candidate for the next–generation detection strategy, electrochemical immunosensor has been intensively studied for its high sensitivity, low cost, excellent detection limits, fast response, ease of handling and miniaturization. However, despite extensive relative researches have been put forward^12^,^13^,^14^,^15^,^16^, developing high performance AFP immunosensor sensor with good sensitivity and selectivity is still necessary.

Recently, label–free electrochemical immunosensor has attracted extensive attention because its inherent property of directly monitoring the binding process of antibody–antigen reaction, avoiding disturbance from conjugated markers, and etc^17^. Due to absent of crosslinking agent, how to efficiently and effectively immobilize the bimolecular is the key factor for a label–free electrochemical immunosensor. Thus the requirements of the electrode materials in label–free electrochemical immunosensor are very strict: excellent carriers in order not to hinder the electron transfer, active sites to attach proteins, and large specific surface area to immobilize more antibodies electrode materials. As a kind of well–known bio–nanomaterial, noble metal can not only well meet the above requirements, but also has some additional advantages, such as well biological compatibility. For example, Wang et al. fabricated a label–free immunosensor based on Pd nanoplates which can detect AFP with a concentration from 0.01 to 75 ng/mL^17^. Huang et al. used GNPs/graphene composite to develop a label–free immunosensor, which showed a linear range of 1 to 250 ng/mL AFP concentrations and could detect AFP in human serum^18^. Xu et al. used the GNPs and enzyme for the detection of AFP, which current varied linearly from 15 to 350 ng/mL of AFP concentration and with a detection limit of 5 ng/mL^6^. However, the existing works almost all focused on the NPs structure of noble metal. As we know, one–dimensional (1D) structure possessed larger specific surface area and better electronic transmission ability compared with NPs materials, these characteristics make it can facilitate the electron transfer process more directly and immobilize the biomolecule more effectively, consequently improve the performance of the sensors.

Accordingly, we developed a series label–free electrochemical immunosensors for amperometric immunoassay of AFP using GNRs with different aspect ratio and compared with that of GNPs. Due to the positively charged surface, the GNRs can absorb on the surface of glassy carbon electrode (GCE) after activation through electrostatic adsorption, and do not need to use nafion or chitosan as adhesion agent which has poor conductivity. On the other hand, it can also directly immobilize matrix for the antibodies or antigens without the use of enzymes or the participation of crosslinking agent due to the positively charged of surface CTAB. This method can greatly simplify the experimental steps. Moreover, the large specific surface area, good electrical conductivity, strong adsorption ability, and improved biological compatibility make the proposed GNRs modified AFP immunoassay showed good performance^19^,^20^, such as high sensitivity, low detection limit, wide detection range, the promoted stability, and moreover, good accuracy to detect AFP in real serum.

## Results and discussion

### Morphological characterization of GNRs and GNPs

**Figure 1:**
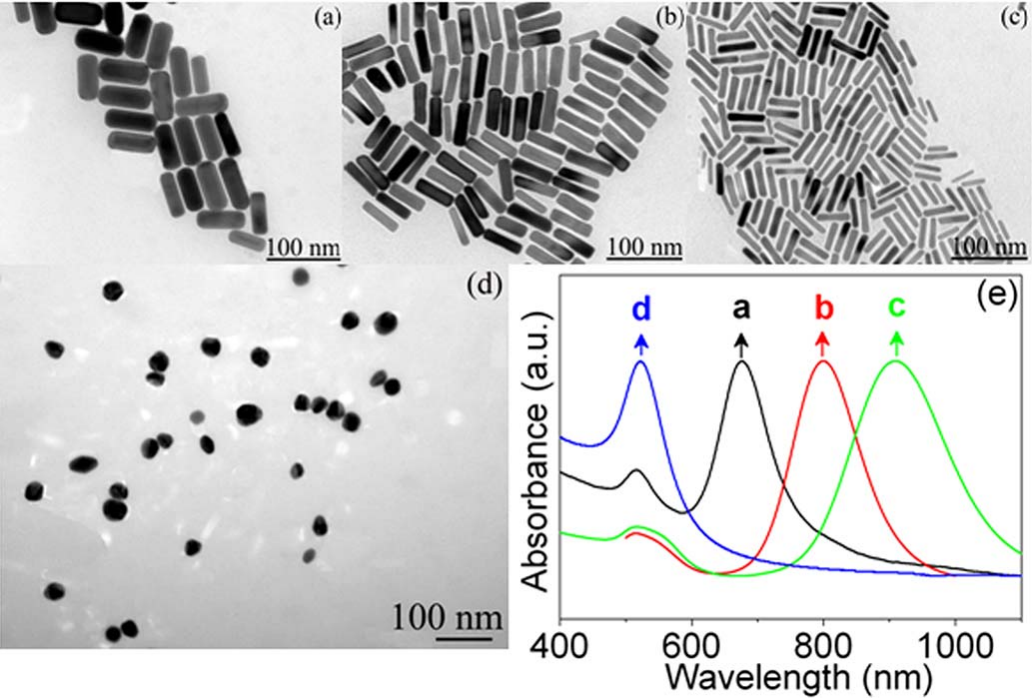
TEM images of (a) GNRs1, (b) GNRs2, (c) GNRs3, and (d) GNPs1. (e) The absorption spectra of the GNRs and GNPs corresponding to (a-d) in TEM images, respectively.

Fig. 1a-c display the typical transmission electron microscopy (TEM) images of as–fabricated GNRs with different amount of initial Au seed corresponding to GNRs1 to GNRs3, respectively. As is shown, different kinds of uniform and densely GNRs can be observed in all the synthetic nanomaterials. The length of GNRs is almost the same (~ 67 nm), while the width is gradually decreased from GNRs1 to GNRs3, 28 nm, 16 nm, and 9 nm, respectively. That is to say, their aspect ratio is ~2.4, 3.7 and 4.8 corresponding to GNRs1 to GNRs3, respectively, which will result in the different specific surface area. Generally, in seed–mediated growth method as used in this work, the gold seed serve as the active sites on which to grow more anisotropic nanostructures and no metal salts could be reduced to metal unless the seeds are present^21^. When these seeds are added to a solution containing more metal salt, a weak reducing agent (ascorbic acid, AA), and a rodlike micellar template (CTAB), the seeds will serve nucleation sites for NRs growth. In this process, CTAB has a tendency to form elongated rodlike micellar structures that possibly assist in rod formation, as well as enhances the rod yield, however, concentrated CTAB can only let GNRs grow within a certain length range (aspect ratio<7), repeating to add growth solution that containing HAuCl_4_ and CTAB is necessary to obtain GNRs with high aspect ratio (>7)^21^. In our case, the seed solution is the only variable, that is to say, the more the seed solutions are added, the more nucleation sites are obtained. Under a certain concentration of HAuCl_4_, the solution with less nucleation sites will possess much more metal salt source to assemble on the minor axis of GNRs, that’s why the aspect ratio growth in the way of diameter decreased while length is almost the same in our case. Besides, Fig. 1d shows the morphology of GNPs which are designated as reference samples, revealing that the GNPs are also uniformly disperse with diameter of ~30 nm, which is comparable with the width of GNRs1 ( ~28 nm).

In addition, The SEM images of GNRs1 modified GCE were also provided in Fig. S1. As can be seen, the GNRs1 were uniformly and closely packed together after modified on the GCEs. Because the surface of GNRs is full of CTAB which make the GNRs are positively charged^22^,^23^, and the GCE is fully sulfuric acid activation before modified. It has been proved that the surface of electrode could generate oxygen–containing functional groups (such as carboxyl, carbonyl, hydroxyl, etc.) after sulfuric acid activation which is negatively charged^24^,^25^,^26^. So the positively charged surface of the GNRs can adsorb on the negatively charged surface of GCE after activation through electrostatic adsorption, and do not need to use nafion or chitosan as adhesion agent which has poor conductivity.

The ultraviolet–visible (UV–Vis) absorption spectra of three kinds of GNRs compared with GNPs were further conducted, as shown in Fig. 1e. The absorption peak of GNPs shows conventional plasmon band around 520 nm. As the aspect ratio increasing, this short wavelength plasmon band becomes very weak, and an additional longitudinal plasmon band appeared. It is clearly that the absorption peak of longitudinal plasmon resonance has an obviously shift from 600 to 950 nm corresponding to GNR1 to GNR3, indicating that the effective increase of the length to diameter ratio from GNR1 to GNR3^22^,^23^.

### Electrochemical characterization of the immunosensor

**Figure 2:**
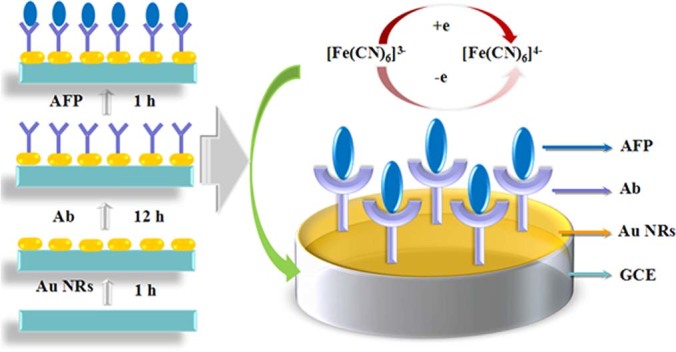
The preparation process and reaction mechanism of GNRs modified immunosensor to the detection of AFP.

**Figure 3:**
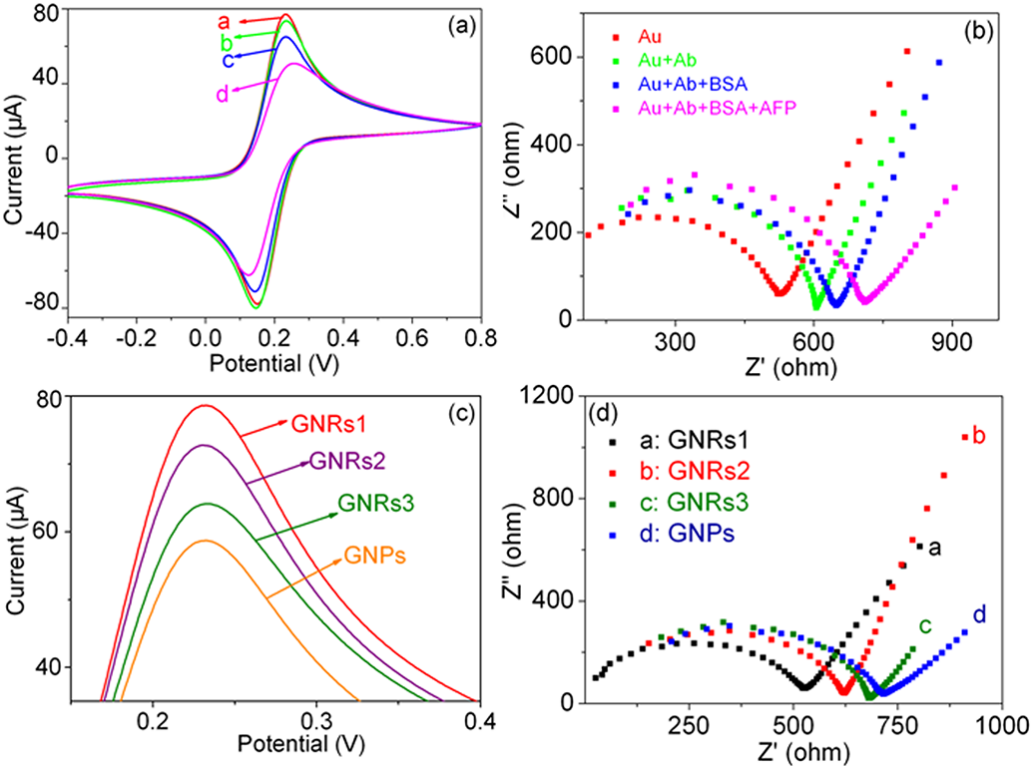
(a) CVs and (b) EIS spectra of the fabrication progress of the GNRs1 modified immunosensor in pH 7.0 PBS containing 5.0 mM Fe(CN)6^3−^/^4−^. a: GNRs1/GCE; b: Ab/GNRs1/GCE; c: BSA/Ab/GNRs1/GCE; d: AFP/BSA/Ab/GNRs1/GCE. (c) CVs and (d) EIS spectra of the GNRs1-GNRs3 and GNPs modified electrodes. a: GNRs1/GCE; b: GNRs2/GCE; c: GNRs3/GCE; and d: GNPs/GCE.

The stepwise self–assemble process of preparing the GNRs modified AFP immunosensor was simulated in Fig. 2. The cyclic voltammetry experiments (CVs) were first employed as a marker to characterize the fabrication process of the immunosensor through monitoring the electrode behavior. As shown in Fig. 3a, when the bare GCE was immobilized with GNRs1 (GNRs1/GCE, curve a), an electrochemical signal with the highest peak current was obtained. Then, a decreased current response is observed after the loading of Ab (Ab/GNRs1/GCE, curve b). This is because the successful immobilization of Ab on the surface of GNRs1/GCE which could form an electron–blocking layer and hinder the efficiency electron transfer. Subsequently, the current response is further decreased after being blocked with bovine serum albumin (BSA, BSA/Ab/GNRs1/GCE, curve c) and then incubated in a solution contained 20 ng/mL AFP (AFP/BSA/Ab/GNRs1/GCE, curve d), which can be ascribed to the insulating protein layers on the electrode further retarding the electron transfer. The whole CV experiment indicates that the fabrication process of the immunosensor is successful.

Electrochemical impedance spectroscopy (EIS) can give the information about the stepwise assembly of the immunosensor from the view of impedance changes of the electrode surface. In EIS, the semicircle diameter equals the electron–transfer resistance (R_et_). Fig. 3b shows the Nyquist plots of different modified electrodes in the presence of redox probe [Fe(CN)_6_]^3−/4−^ and curves a to d represent the same steps as presented in Fig. 3a. Curve a represents the resistance of the electrode after deposition of GNRs1. From the curve b, c and d of Fig. 3b, we can see that after the Ab, BSA and AFP were incorporated, respectively, R_et_ gradually increased with the fabrication process of the immunosensor. The reason is that the immunocomplex layer on the electrode acts as the electron communication and mass–transfer blocking layer, further insulating the conductive support and hindering the access of redox probe toward the electrode surface significantly, thus, a further increase of the resistance is observed. The gradually EIS change of the modified process proves that the Ab, BSA and AFP are all successfully immobilized on the surface of GNRs1/GCE electrode, which is in good agreement with the CV results in Fig. 3a.

**Figure 4:**
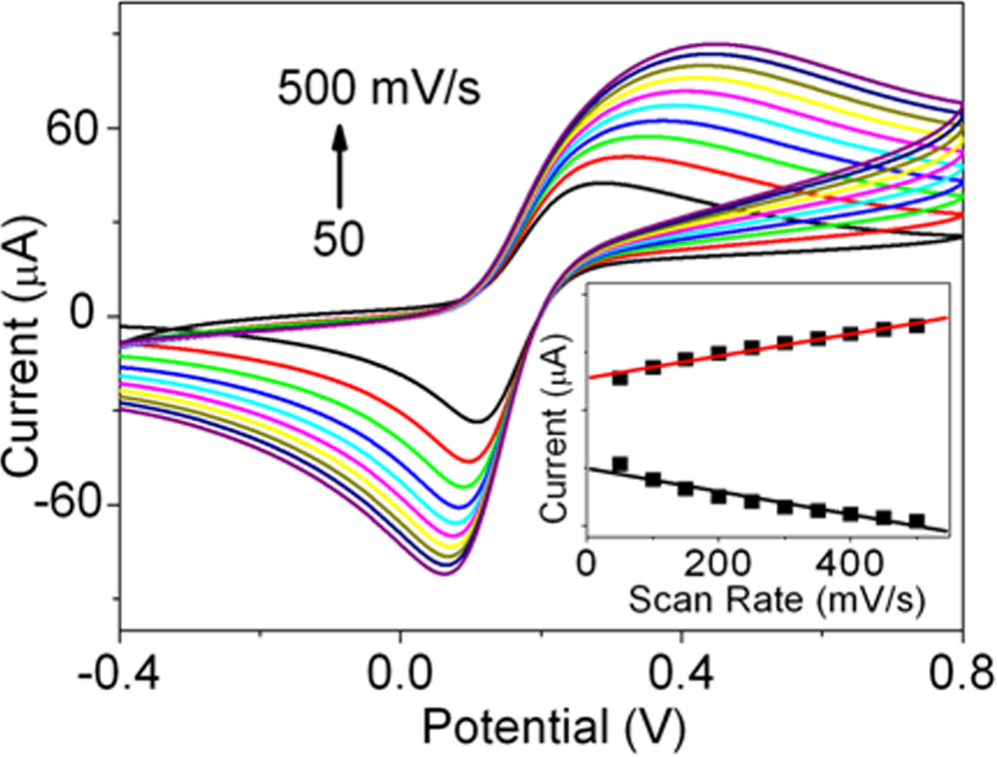
CVs of the GNRs1 modified immunosensor with 20 ng/mL AFP in pH 7.0 PBS containing 5.0 mM Fe(CN)_6_^3−^/^4−^ at different scan rate of (from a to j): 50, 100, 150, 200, 250, 300, 350, 400, 450, and 500 mV/s, respectively. The inset shows the linear relationship between the peak currents and the square root of scan rate.

To observe the effect of different aspect ratio of GNRs, the intensities of electrochemical signal were compared when the bare GCEs were immobilized with different GNRs. As shown in Fig. 3c, the intensity gradually decreases with the decreasing of GNRs diameter (from GNR1 to GNR3), indicating that GNR1 possesses the best electronic conductivity. The current intensity of GNPs is the lowest one. In addition, the EIS spectra of different GNRs modified electrodes compared with that of GNPs were also studied. As shown in Fig. 3d, the R_et_ gradually increases with the decrease of the width of GNRs (From GNRs1 to GNRs3), indicating the GNRs1 possesses the best electron–transfer ability in the GNRs. For GNPs modified immunosensor, it also shows the largest R_et_. These facts are because of the restriction of the inherent nanostructure of NPs and the block of surface CTAB. The typical CV curves of the GNR1 modified immunosensor in 0.1 M PBS buffer solution at different scan rates ranging from 50 to 500 mVs^−1^ were studied in Fig. 4, which suggesting a quasi–reversible diffusion–controlled behavior with an electron transfer process. As is observed in Fig. 4, a pair of roughly symmetric anodic and cathodic peaks appears with almost equal peak currents in the studied scan rate range, and the peak–to–peak separation also increases with the scan rate. The anodic and cathodic peak currents both are linearly proportional to the square root of the relevant scan rate (inset of Fig. 4), suggesting a quasi–reversible diffusion–controlled behavior with an electron transfer process.

### Optimization of detection conditions

**Figure 5:**
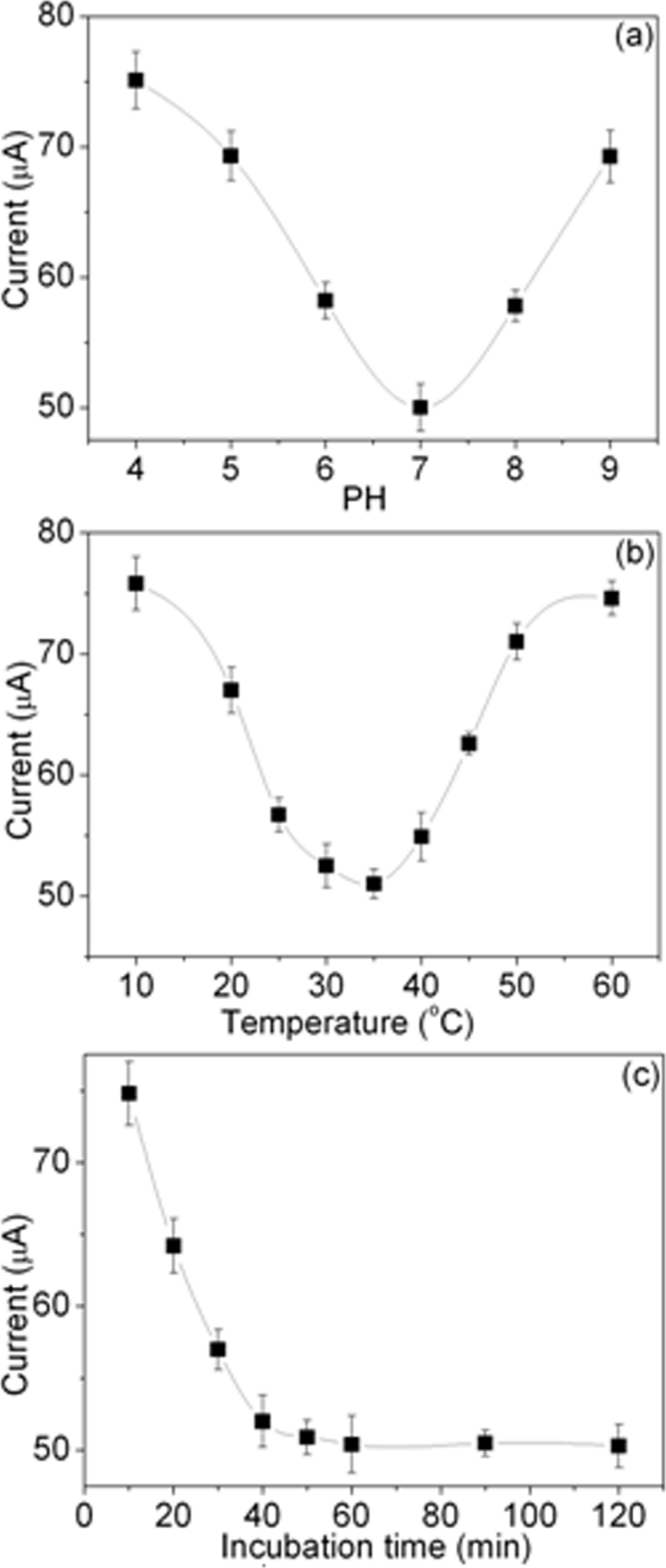
Effects of (a) pH value, (b) incubation temperature, and (c) incubation time on the current responses of the developed immunosensor to 20 ng/mL AFP.

The performance of the immunosensor is mainly influenced by pH value, incubation temperature and incubation time, thus the detection conditions should be carefully optimized. Fig. 5a shows the dependence of the electrochemical behavior of the immunosensor incubated in 20 ng/mL AFP solution in the pH ranging from 4.0 to 9.0 in PBS solution containing 5 mM K_3_Fe(CN)_6_. The amperometric response of immunosensor decreases along with the increasing pH value from 4.0 to 7.0 and then the response increases with further increasing pH value. That is because highly acidic or alkaline surroundings would damage the immobilized protein stability and activity. Thus, pH 7.0 was selected as the optimum pH value for AFP antigen detection.

Temperature effect of the immunosensor was studied in PBS (pH 7.0) solution containing 5 mM K_3_Fe(CN)_6_ using 20 ng/mL AFP with incubation temperature range from 10°C to 60°C. As shown in Fig. 5b, it is found that the peak current response gradually decreases when the temperature increases from 10°C to 35°C and then begins to increase when the temperature surpassed 35°C. The reason may be attributed to the fact that during the temperature increases from 10°C to 35°C, numerous immunocomplex are formed and inhibit the current response, whereas higher temperature above 40°C may cause irreversible denaturation of AFP and anti–AFP involved in the process. In consequence, 35°C is an optimal temperature of immunoreaction. However, considering the activity of biomoleculars and the life–time of biosensor, the 37°C is used as incubation temperature.

The effect of the incubation time on the amperometric response (when the 20 ng/mL AFP connected to the antigen reaction occurs) was also investigated. In the incubation solution, when the antigens reach the antibodies on the electrode surface of the immunosensor, it takes some time for the contacting species to form immunocomplexes, and the results are shown in Fig. 5c. With increasing incubation time, the current responses decrease and then stabilize when the incubation time is longer than 60 min. The flat shows that combined capacity of the antigen on the sensor gradually tends to saturation. Thus, an incubation time of 60 min is adopted in the subsequent work.

### Analytical performance of the immunosensor

**Figure 6:**
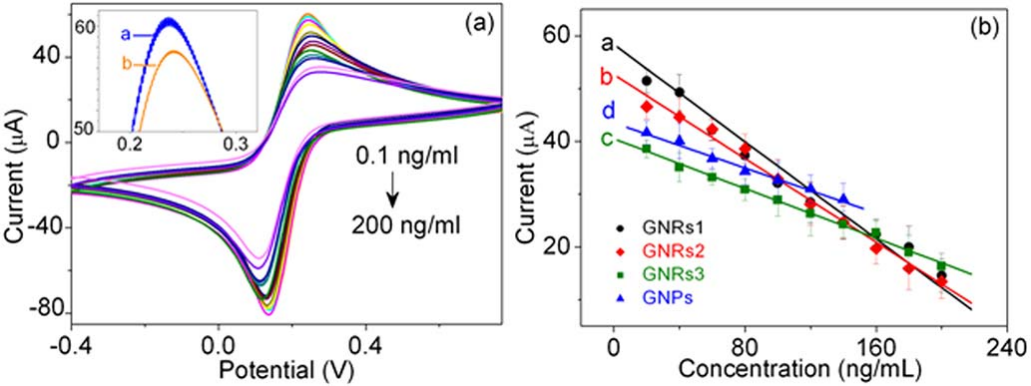
(a) The CVs of the GNRs1 modified immunosensor at different concentrations of AFP in human serum. (b) The calibration curves of different proposed immunosensors to different concentrations of AFP.

Under the optimized detection conditions, the sensitivity and dynamic range of the electrochemical immunosensor were evaluated toward the CV measurement to detect AFP in 0.1M PBS (pH 7.4) containing 5 mM K_3_Fe(CN)_6_. First, the GNRs1 modified immunosensor was exposed to various concentrations of AFP solutions and performed at least triplicate analyses at each concentration using the proposed immunosensor. As shown in Fig. 6a, it can be found that the current signal decreases gradually with increasing concentration of AFP. That because more and more AFP could bind to the immobilized antibodies at higher concentrations of antigens, and the antigen–Ab complex acts as an inert kinetic barrier for the electron–transfer of the mediator of ferricyanide. Besides, as depicted inset of Fig. 6a, it can be seen that after dropped with 0.1 ng/mL AFP, the peak current of curve b clearly decreased compared with the unincubated one (curve a). Since the threshold value of the AFP in normal human serum is ~10 ng/mL^27^, as developed immunosensor could meet the requirement of practical application in clinical immunoassay.

In addition, the calibration plots of the developed immunosensor based on different GNRs were also studied and compared with that of GNPs, as shown in Fig. 6b. All the calibration plots exhibited a good linear relationship in the studied range. The corresponding linear equation, correlation coefficient, linear range, and detection limit of each developed immunosensor are summarized in Table 1. As is listed, the slopes of the calibration curves based on GNRs (curve a–c) decrease with the reduced of the diameter of GNRs (from 28 nm to 9 nm, corresponding to GNR1 to GNR3), that is to say, the bigger the diameter in our case (length is almost the same), the higher the sensitivity. Moreover, the current change of developed immunosensor based on GNRs1 is linear proportional to AFP concentration in the range of 0.1–200 ng/mL with a detection limit of 0.04 ng/mL (S/N = 3). When the AFP level is higher than 200 ng/mL, an appropriate dilution should be preferable. Besides, we also fabricated the GNPs which diameters (~ 30 nm, curve d) are comparable with the width of GNRs1 (~ 28 nm). The linearity range of GNPs modified immunosensor is much narrow and also has a stronger back ground current, although the GNPs modified immunosensor seems achieve a larger linear slope, therefore, it does not show better advantages. Table 2 shows a comparison of the GNRs1 modified immunosensor in this work with other noble metal modified AFP immunosensors. It can be seen that the linear range of GNRs1 modified immunosensor is satisfactory, and the detection limit of GNRs1 modified immunosensor is much lower than other immunosensors, excepted the Au and prussian blue modified ITO immunosensor which showed the same detection limit and Pb nanoplates immunosensor which showed a lower limit. However, the preparation method of that immunosensor was more complicated and the Pb nanoplates immunosensor had a much narrower linear range. The good electrochemical performance of the immunosensor based on GNRs were mainly attributed to GNRs possess good electron transfer capability and larger specific surface area which can be capped more CTAB, especially the GNR1 one which possess larger diameter since the length is almost the same, and then greatly enhanced the electrochemical signal.

### Selectivity, stability and reproducibility of the immunosensor

**Figure 7:**
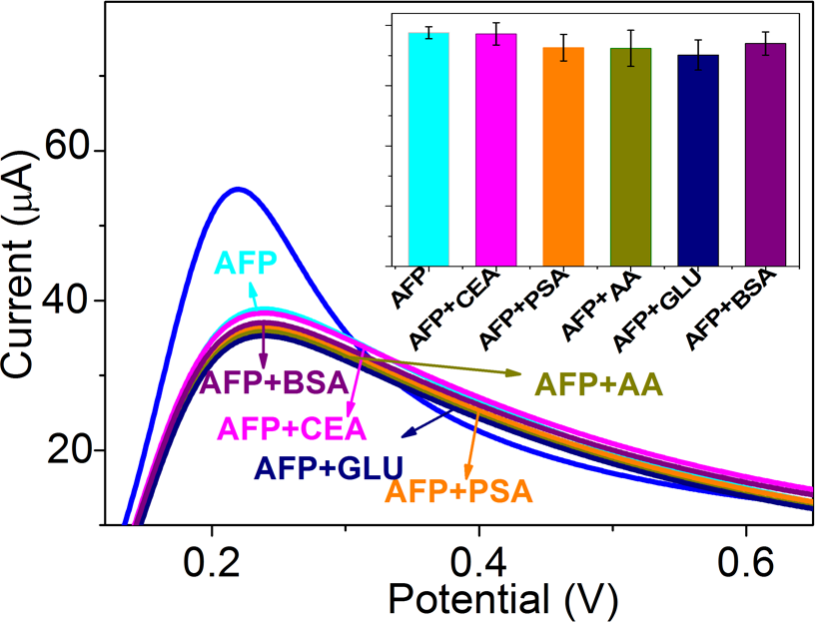
The specificity of the GNRs1 modified immunosensor toward AFP, AFP+CEA, AFP+PSA, AFP+AA, AFP+glucose, and AFP+BSA. Inset is the corresponding histogram.

Since the immunosensor based on GNRs1 exhibited the best electrochemical performance to AFP, further researches were conducted on it. To monitor the response of the proposed immunosensor to interference degree or crossing recognition level, some possible interfering agents that potentially co–existed in human serum, such as embryonic antigen (CEA), prostate specific antigen (PSA), AA, BSA, and glucose were used to evaluate the specificity of the GNR1 modified immunosensor, as shown in Fig. 7. The immunosensor was incubated with 20 ng/mL AFP in the presence of 100 ng/mL interfering agents. Compared with the current response caused by pure AFP, the variation in current caused by the interfering substances was less than 10%, even in the condition that the concentration of the interfering agents is 5 times higher than that of AFP, indicating the high specificity of the proposed electrochemical immunosensor.

**Figure 8:**
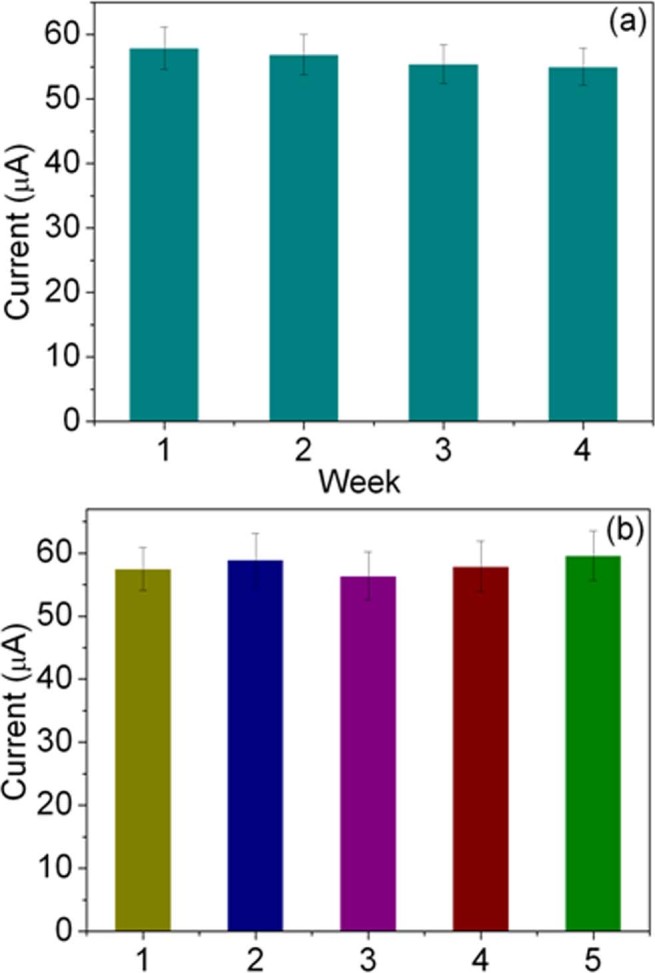
(a) The stability of the electrochemical immunosensor. The current response of the electrodes was observed every week until the forth week and the current value is stable. (b) The repeatability of the GNRs1 modified immunosensor was evaluated from the response to 20 ng/mL AFP at five different electrodes.

The stability of the immunosensor was also tested, as shown in Fig. 8a. When the immunosensor was not in use, it was stored in PBS (pH 7.4) at 4°C. The current response of the electrodes only had a change of 1.7% after 2 weeks, and a 5.2% decrease of the initial response was observed after 4 weeks. This indicates the effective retention of the activity of the immobilized AFP antibody. The reproducibility is an important feature to check the reliability of the developed immunosenor. It was investigated by using the variation coefficients of interassays, which was evaluated by analysis of the same concentration of AFP (10 ng/mL) using five equally prepared electrodes. As shown in Fig. 8b, five independently made immunosensors exhibited similar current response and the interassay variation coefficient of interassays is 5.5%. The low relative standard deviation indicates that the proposed immunosensor displayed good repeatability.

### Analysis of clinical serum samples

**Figure 9:**
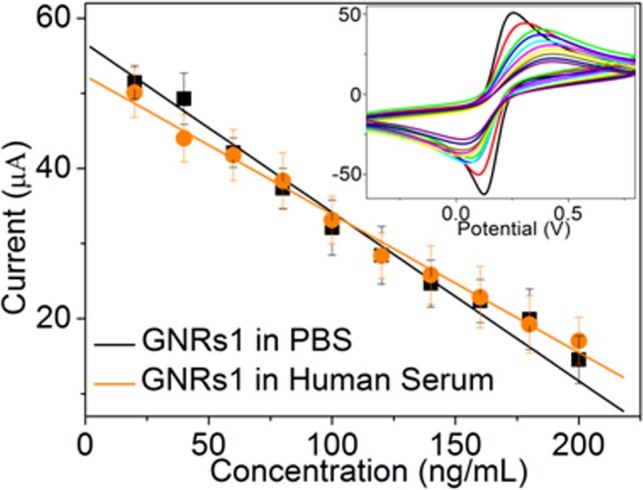
The calibration curves of GNRs1 modified immunosensor to different concentrations of AFP in PBS solution and in human serum. (Inset is the corresponding CV curves in human serum)

To monitor the analytical reliability and possible application of the developed immunosensor, the immunosensor based on GNRs1 was used for the determination of the concentration of AFP in human serum compared to the concentration of AFP in PBS buffer solution, which was shown in Fig. 9. Inset is the cyclic voltammograms of the GNRs1 modified immunosensor at different concentrations of AFP in human serum. The slope of the two curves in Fig. 9 changed little. The results are then compared with the reference values obtained by the ELISA. As summarized in Table 3, there is no significant difference between the two methods, indicating that the developed immunoassay methodology could be potentially useful for determination of AFP in biological samples.

## Conclusions

In this work, GNRs with different aspect ratios have been successfully synthesized by using a wet–chimcal method, where the simultaneously generated positively charged CTAB on the surface. Compared to GNPs, GNRs possess lager electro–active surface area that can conjugated with Ab which can specifically link to the AFP antigen, and higher electrical conductivity which can promote electron transfer reactions. The as proposed GNRs modified immunosensors exhibited good electrochemical response to AFP which showed much larger linear range and low detection limited compared to GNRs modified immunosensor. The GNRs1 modified immunosensors is the best one with the detection limit of 0.04 ng/mL for the AFP antigen, good stability and reproductivity. Moreover, it also could accurate detection of AFP in human serum. This label–free preparation method was simple and sensitive for determination of AFP with good precision and accuracy, which can be further developed for other immunoassays.

## Materials and methods

### Materials and apparatus

The AFP, Ab, CEA, and PSA were purchased from Beijing Boisynthese Biotechnology Co., Ltd. (Beijing, China). BSA (96–99%) was purchased from Beijing DingGuo Biotechnology Company. Human serum samples were purchased from a local hospital. All the other chemicals were of analytical reagents grade and used without further purification. 5 mM K3[Fe(CN)6] was used as electrolyte for all electrochemistry measurement.

TEM images were recorded on JEM-2010 transmission electron microscope under a working voltage of 200 kV. UV-Vis absorption spectra were measured with Shimadzu UV-3101PC UV-vis scanning spectrophotometer ranging of 200–1100 nm. All experiments were conducted using a three-electrode electrochemical cell with a glassy carbon based working electrode, a calomel reference electrode, and a platinum wire counter electrode. Note that all electrochemical measurements were performed in PBS (pH 7.0) solution containing 5 mM K3Fe(CN)6. Electrochemical measurements were performed on a model CHI630D electrochemical analyzer (ChenHua Instruments Co. Ltd., Shanghai, China). EIS was performed on a model CHI660D electrochemical analyzer (ChenHua Instruments Co. Ltd., Shanghai, China).

### Preparation of GNRs and GNPs

The GNRs and GNPs were fabricated according to the seed-mediated growth method by EI-Sayed and co-workers^28^, which could be divided into two steps. First is the seed-mediated growth procedure. The gold seed solution was prepared by reduction of HAuCl_4_ (2.5 mL, 1 mM) with NaBH_4_ (0.6 mL, 0.01 M) after ultrasonic in the presence of CTAB (7.5 mL, 0.1 M) after dissolving in the oven. After 2 hrs of reaction, the preparation of seed solution was completed. In second step, 66 mL of 0.1 M CTAB was mixed with 60 mL of 1 mM HAuCl_4_ in a flask and the flask was put into the oven for 30 min to dissolve, and then 1.2 mL of 10 mM silver nitrate aqueous solution and 1.1 mL of 2 M hydrochloric acid were added into the flask. After gentle mixing of the solution, AA (0.96 mL, 0.1 M) was added with continuous stirring, and then the seed solution with different amount was finally added into the mixture quickly to get GNRs with different length to diameter ratio. The GNRs were then aged 5 hrs and the GNPs were aged 12 hrs at 28°C water bath to ensure full growth. After that, excess CTAB molecules were removed by centrifuging twice at 12 000 rpm for 15 min, and then redispersed in 2 mL ultrapure water.

### Fabrication of the GNRs modified AFP immunosensor

Before surface modification, the GCE (dia. 3 mm) used to consist of immunosensors were carefully polished with 0.1 and 0.05 µm Al_2_O_3_ powder, respectively, to a mirror-like surface, then ultrasounded sequentially in dilute nitric acid (0.5 M), acetone and ethanol for 2.5 min. Then the GCE electrodes were activated in sulfuric acid (0.5 M) for 2 hrs to further clean the electrodes and get some oxygen containing functional groups on its surface, such as carboxyl, carbonyl, hydroxyl, etc^29^. First, 5 µL of the GNRs aqueous solution (0.2 mg/mL) was dropped on the surface of GCE (Au/GCE) with oxygen containing functional groups and waited to dry, and the GNRs were adsorbed on the surface of electrode due to electrostatic adsorption. Second, 10 μL of Ab solution was dropped on the Au/GCE for 12 hrs at 4°C to anchor the antibody on the surface of the electrode and to form Ab/Au/GCE. Third, the Ab/Au/GCE was immersed in 5 wt% BSA solution for 1 h at room temperature to block possible remaining nonspecific binding of Ab/Au/GCEs active sites. Finally, 10 μL of AFP with different concentration from 0.1 to 200 ng/mL was dipped onto the electrode for linking with the anti-AFP antibody and wait for about 1h to firmly fixed on the electrode. For comparison, GNPs modified AFP immunosensor was also prepared in the same way.

## Author Contributions

C.Y.Z. conducted the most of investigation for the samples and wrote the main paper. L.X. supervised the project, had given valuable advices on the proceeding of this work, and revised the manuscript. J.S. Q.L.L. D.L.L. and H.W.S. had provided precious suggestions on the selection of test and analysis of experimental data about electrospinning. R.Q.X. and S.X. supported the characterization of the samples. All authors discussed the results and commented on the manuscript at all stages.

## Supplementary Material

Supplementary Information A sensitive label-free amperometric immunosensor for alpha-fetoprotein based on gold nanorods with different aspect ratio

## Figures and Tables

**Table 1:** A list of linear equation, correlation coefficient, linear range, and detection limit of each developed immunosensor in our work.

Immunosensor	Linear Equation	Correlation Coefficient	Linear range (ng/mL)	Detection limit ( ng/mL )
GNRs1	Y_1_ = −0.22x+55.6	R_1_^2^ = 0.988	0.1–200 ng/mL	0.04 ng/mL
GNRs2	Y_2_ = −0.19x+52.6	R_2_^2^ = 0.990	0.1–200 ng/mL	0.06 ng/mL
GNRs3	Y_3_ = −0.12x+40.4	R_3_^2^ = 0.994	0.1–200 ng/mL	0.07 ng/mL
GNPs	Y_4_ = −0.12x+54.2	R_4_^2^ = 0.990	5–140 ng/mL	0.13 ng/mL

**Table 2:** Comparison of analytical properties of the developed immunoassay with other noble metal modified AFP immunosensors.

Analysis of materials	Electrode material	Linear range	Detection limit	Ref.
GNRs1 modified immunoassay	GCE	0.1–200 ng/mL	0.04 ng/mL	Our Work
Au NPs/carbon nanotube/chitosan	GCE	1–55 ng/mL	0.6 ng/mL	5
Self–assembled monolayers AuNPs/HRP	Silicon wafer	15–350 ng/mL	5 ng/mL	6
AuNPs/TiO_2_ NPs/chitsotan	GCE	1–160 ng/mL	0.1 ng/mL	12
Pb nanoplates	GCE	0.01–75 ng/mL	0.004 ng/mL	17
AuNPs/Graphene	Carbon ionic liquid electrodes	1–250 ng/mL	0.1 ng/mL	18
AuNPs–Ab/AFP/Ab–HRP sandwich structure	GCE	5–80 ng/mL	3.7 ng/mL	30
Au and prussian blue	Indium tin oxide	0.25–300 ng/mL	0.04 ng/mL	31
Au NWs/ZnO NRs/GCE	GCE	0.5–160 ng/mL	0.1 ng/mL	32
AuNPs/Polyamidoaminic dendrimers	GCE	15–500 ng/mL	3 ng/mL	33

**Table 3:** Comparison of AFP levels determined using proposed method and ELISA.

Serum Samples	Our Work	ELISA	Relative deviation (%)
1	3.21	3.44	7.2
2	4.19	4.31	2.9
3	5.93	5.76	2.9
4	7.38	7.16	3
5	10.8	11.2	3.7
