# Supplementary material for: A sensitive label–free amperometric immunosensor for alpha-fetoprotein based on gold nanorods with different aspect ratio
Source: Sci Rep. 2015 Apr 24;5:9939. doi: 10.1038/srep09939 (PMC4408978; doi:10.1038/srep09939)
Supplement: Supplementary Information — A sensitive label-free amperometric immunosensor for alpha-fetoprotein based on gold nanorods with different aspect ratio [file srep09939-s1.doc]

**A sensitive label–free amperometric immunosensor for alpha-fetoprotein based on gold nanorods with different aspect ratio**

Chunyang Zhou,^†^ Dali Liu,^†^ Lin Xu*,^†^ Qingling Li,^†^ Jian Song,^†^ Sai Xu,^†^ Ruiqing Xing,^†^ Hongwei Song*^†‡^

*^†^State Key Laboratory on Integrated Optoelectronics, College of Electronic Science and Engineering, Jilin University, 2699 Qianjin Street, Changchun, 130012, P. R. China.*

*^‡^The State Key Laboratory of Bioelectronics, Southeast University, 210096 P. R. China.*

*
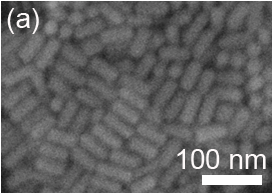

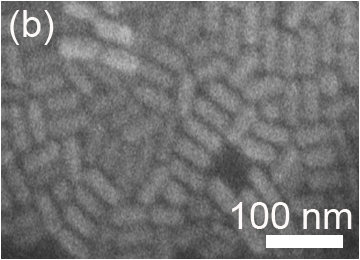
*

Fig. S1. The SEM images of GCE modified with GNRs1.

As can been seen the GNRs1 were uniformly and closely packed together after modified on the GCEs due to the electrostatic adsorption.
